# Safety Assessment of Prolonged Nerve Catheters in Pediatric Trauma Patients: A Case Series Study

**DOI:** 10.3390/children11020251

**Published:** 2024-02-16

**Authors:** Nicole Verdecchia, Alexander Praslick, Mihaela Visoiu

**Affiliations:** Children’s Hospital of Pittsburgh, University of Pittsburgh Medical Center, 4401 Penn Ave., Pittsburgh, PA 15224, USAvisioum@upmc.edu (M.V.)

**Keywords:** prolonged nerve block, infection risk, regional anesthesia, pediatric regional anesthesia, acute pain, trauma

## Abstract

Introduction: Nerve block catheters (NBCs) are increasingly used for pain management in pediatric trauma patients. While short-term efficacy has been well established, the long-term safety of NBCs is unknown. Methods/Cases: The retrospective chart review includes a cohort of nine pediatric trauma patients aged 3–15 years who received 52 peripheral nerve block catheters and epidurals for pain management. This study aimed to investigate the potential risks associated with the prolonged use of NBCs in pediatric trauma cases. Results: The NBCs (48 peripheral catheters and 4 epidural catheters) were maintained for about 2 weeks. The number of catheters per patient varied from 1 to 11. The study noted a low frequency of catheter-related complications. No catheter-site infection or local anesthetic toxicity symptoms were reported. Discussion: These findings suggest that NBCs can be safely maintained for extended periods in pediatric trauma patients without significantly increasing complications. Careful monitoring and adherence to infection control practices remain paramount when implementing extended catheter use.

## 1. Introduction 

Pain control in children is critical since undertreated pain can lead to long-term medical and psychiatric consequences [1]. One way to manage pain in the trauma patient is regional anesthesia. However, many trauma patients require repeated surgeries, and pain control remains an issue for weeks. Risks with performing prolonged regional anesthesia catheters include the risk of infection, local anesthetic toxicity, and nerve injury. Walker and colleagues did not find any incidence of local anesthetic toxicity or prolonged nerve injury in pediatric patients who received peripheral nerve catheters [2]. 

In a study of 2074 pediatric patients with peripheral nerve catheters, Walker and colleagues found an infection rate of 0.9%. The only significant predictor of infection these authors found was catheter duration: a median (IQR) of 4.5 days (3–7) for infected catheters compared to 3 (1–3) days for non-infected catheters [2]. It is standard to remove peripheral nerve catheters after 48–72 h (about 3 days) since data show the infection rate increases at this duration, although infection is still rare [3,4,5,6]. However, in certain situations, longer catheter durations have been reported, including trauma and cancer patients. There is a case report of a pediatric trauma patient having a popliteal nerve catheter for 46 days (about 1 and a half months) [7]. Another case series reported placing continuous nerve catheters for 22–36 days (about 1 month 5 and a half days) in four different patients for pathologic fractures due to cancer pain [8]. Another case report describes a continuous popliteal catheter for 68 days (about 2 months 1 week) in a military combat-related polytrauma requiring multiple orthopedic surgeries [9].

Infection in continuous peripheral nerve blocks is, fortunately, a rare event. It has been reported that colonization of the catheter tip with skin microbes is common, with rates up to 50% [3,10,11]. However, the risk of infection leading to abscess formation or requiring antibiotics is low, around 0–3.2% [11,12,13]. The most common sites of infection are femoral and axillary catheter site insertions, but interscalene insertions also have case reports of severe infection [11,12,13,14]. Use of prophylactic antibiotics, site of insertion, aseptic technique, and catheter duration have all been shown to influence the risk of severe infection, while tunneling and the use of bio patch have not been studied and remain controversial [12,14,15,16]. 

We present a case series of nine pediatric trauma patients who received 52 continuous peripheral nerve and epidural catheters for up to 15 days (about 2 weeks) with no infectious complications requiring additional treatment. 

## 2. Cases and Results

The University of Pittsburgh’s Institutional Review Board approved the study, Study number 23050074, on 28 June 2023. Informed consent was waived due to a HIPAA authorization form being signed, and it is also included in anesthesia consent, and all data was deidentified. The ages of the nine patients in this retrospective study ranged from 3 to 12 years. Their injury complex had degloving injuries, avulsions, traumatic amputations, and one case of limb necrosis from thrombosis after extracorporeal mechanical support. The patients were all brought to the operating room for repeated irrigation and debridements, wound vacuum placement and changes, and skin grafting surgeries. Inclusion criteria were pediatric patients that had traumatic injuries requiring multiple operations within the time frame of the study. Patients were excluded if they did not receive any nerve block catheter for longer than 72 h. Demographic data is shown in Table 1. 

All patients had more than one catheter placement and up to four catheters per block location. There were 52 total catheters in the nine patients, 48 were peripheral catheters, and four were epidural catheters. The range of duration of each catheter placement was 2–15 days (about 2 weeks), and each patient had between one and 11 catheters placed. Three patients had bilateral catheters placed at the same time. The range of durations of total catheters per patient was 7–33 days (about 1 month). 

All the patients had clonidine 1–2 mcg/mL and 0.2% ropivacaine in their peripheral nerve block solution, with no evidence of toxicity or side effects from local anesthetic or clonidine. Infusions were run at 0.5 mg/kg/h, and bags were usually changed when the infusions completed, which was once every 1–2 days. All patients received antibiotics before surgery as prophylaxis, and most were on scheduled antibiotics for the duration of the nerve block catheter insertion due to their traumatic injury. The dressings of 24% of catheters included a chlorhexidine gluconate-impregnated disc (Biopatch^®^, Ethicon, Inc., Somerville, NJ, USA), and 5% of the catheters were tunneled. No patients had a nerve block catheter-related infection, although several patients had leukocytosis and fever during the time the catheters were indwelling. No patients had local anesthetic complications. No additional treatment was needed for nerve block-related complications. All blocks were placed under general anesthesia when the patients returned to the operating room for debridement and wound vacuum changes. Data is shown in Table 2. 

Catheter-related complications included leaking (4), dislodgement (2), dressing contamination (5), erythema (1), and disconnection (1), but none of these complications resulted in an infection at the catheter site. 

Patient #1 received two right-sided popliteal catheters for 11 days (about 1 and a half weeks) each and one epidural for 7 days. The first popliteal catheter was replaced on day 11 due to leaking and replaced with a tunneled popliteal catheter. An epidural was later placed for free flap definitive surgery, and the patient was no longer on scheduled antibiotics at that point. There were no nerve block-related complications or infections. 

Patient #2 received four right femoral nerve block catheters for 11, 8, 4, and 10 days (about 1 and a half weeks). The patient received two right popliteal and one right sciatic catheter for 11, 10, and 12 days (about 1 week 5 days), respectively. They had two epidurals that were in place for 8 and 6 days, respectively, where the second catheter was tunneled. During treatment, the patient’s antibiotics were completed, and there was a period when the patient was not on antibiotics. The antibiotic treatment was restarted due to leukocytosis several days later. There were no fevers, and the patient was found to be positive for clostridium difficile. One of the patient’s popliteal catheters had to be redressed due to excoriation and peeling of the dressing, but the catheter insertion site was clean, dry, and intact. 

Patient #3 had three right popliteal catheters and three left popliteal catheters for 3, 8, and 7 days, respectively. These were present simultaneously. They subsequently received two bilateral sciatic catheters for 13 and 10 days (about 1 and a half weeks). The patient had an epidural placed for definitive free flap surgery, which was left in place for 8 days and was tunneled. The second left sciatic catheter was leaking on day five and was redressed. The epidural site had redness on day six and was redressed. There was no concern for site infection or abscess. 

Patient #4 had right-sided upper extremity catheters placed, including two supraclavicular catheters and two infraclavicular catheters left in place for 7, 3, 7, and 2 days, respectively. The second supraclavicular catheter was removed early due to leaking and loose dressing, and the second infraclavicular catheter was removed early for the same reason. No infections were noted. This patient had a fever during the second supraclavicular catheter, but the nerve block was not suspected to be the source. 

Patient #5 received a left popliteal continuous nerve catheter for 11 days (about 1 and a half weeks) with no complications noted. 

Patient #6 received three right popliteal catheters for 3, 6, and 9 days, respectively, and two left popliteal catheters for 11 and 9 days, respectively. These were present simultaneously. One of the right catheters was removed accidentally, one was removed due to an unwitnessed disconnect, and one was removed because of fevers. The nerve block sites were clean, with no signs of infection or abscess. 

Patient #7 received four right femoral catheters for 3, 15, 2, and 3 days, four right sciatic catheters for 3, 7, 13, and 5 days, two right lumbar plexus catheters for 2 and 3 days, and one right popliteal catheter for 9 days. The right popliteal catheter had a Biopatch placed. This patient had catheter replacements several times due to urine and stool contamination of the catheter dressings. The patient was febrile due to a wound infection but no known nerve catheter-related infections. 

Patient #8 received bilateral supraclavicular catheters twice due to necrosis and thrombosis of her limbs after an invasive infection. These were left in for 14 days (about 2 weeks) initially. The second right catheter was in place for 7 days, while the left was removed accidentally after 4 days. While she had wound infections, there were no nerve catheter infections. 

Patient #9 received one left sciatic catheter for 9 days, two left femoral catheters for 9 and 3 days, and one left popliteal catheter for 11 days (about 1 and a half weeks). The sciatic catheter dressing was contaminated by stool, so it was removed to prevent site infection. The popliteal catheter had a disconnect and leaking around the dressing, so it was preemptively removed. 

## 3. Discussion

In the pediatric population, reducing opioids and opioid-related complications, reducing parental and caregiver stress, and reducing the risk of chronic pain is essential. A multimodal approach, including regional anesthesia, is critical to achieving these goals [17,18].

Minimal data has been published on the safety of maintaining long-term peripheral nerve and epidural catheters, and standard recommendations are to remove them after 48–72 h (about 3 days) [2,6,10]. This is based on data where the risk of infection is around 0.9% and starts to increase after 72 h (about 3 days) [2], although no specific cases of systemic infection or abscess are reported. In simple surgical patients, this may be the best recommendation. However, this may not be beneficial in certain situations when patients require multiple surgeries with a prolonged course. The current study revealed that prolonged peripheral nerve catheters can be considered in specific pediatric trauma cases where pain control remains a challenge. Despite prolonged catheter use, the infection rate remained low, with no catheter-related infections or infections at the insertion site. This is comparable to a similar case report of a pediatric trauma patient who maintained a peripheral nerve catheter for 46 days (about 1 and a half months) [7]. 

**Figure 1:**
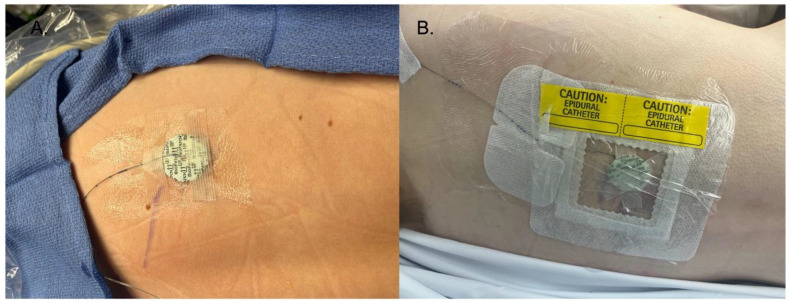
(**A**) The initial dressing of the catheter was surgical skin glue at the insertion site, liquid medical adhesive surrounding the catheter, and the Biopatch on top of the insertion site. Strips of medical tape are then placed on top of the Biopatch. (**B**) Finished dressing, with a transparent catheter securement dressing on top of the steri strips, Biopatch, and catheter, followed by transparent dressings. Finally, the dressing is labeled with the site.

**Figure 2:**
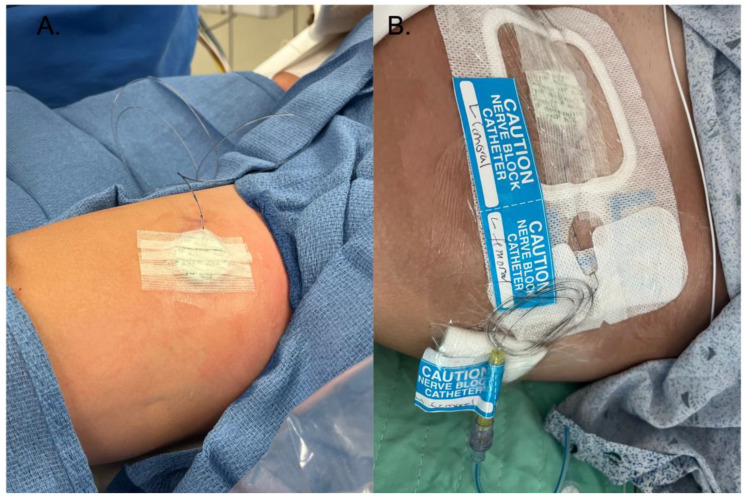
(**A**) This is the first steps of securing the catheter, including surgical skin glue at the insertion site, liquid medical adhesive surrounding the catheter, and the Biopatch on top of the insertion site. Strips of medical tape are then placed on top of the Biopatch. (**B**) Finished dressing, with a transparent catheter securement dressing on top of the steri strips, Biopatch, and catheter, followed by transparent dressings. Finally, the dressing is labeled with the site.

**Figure 3:**
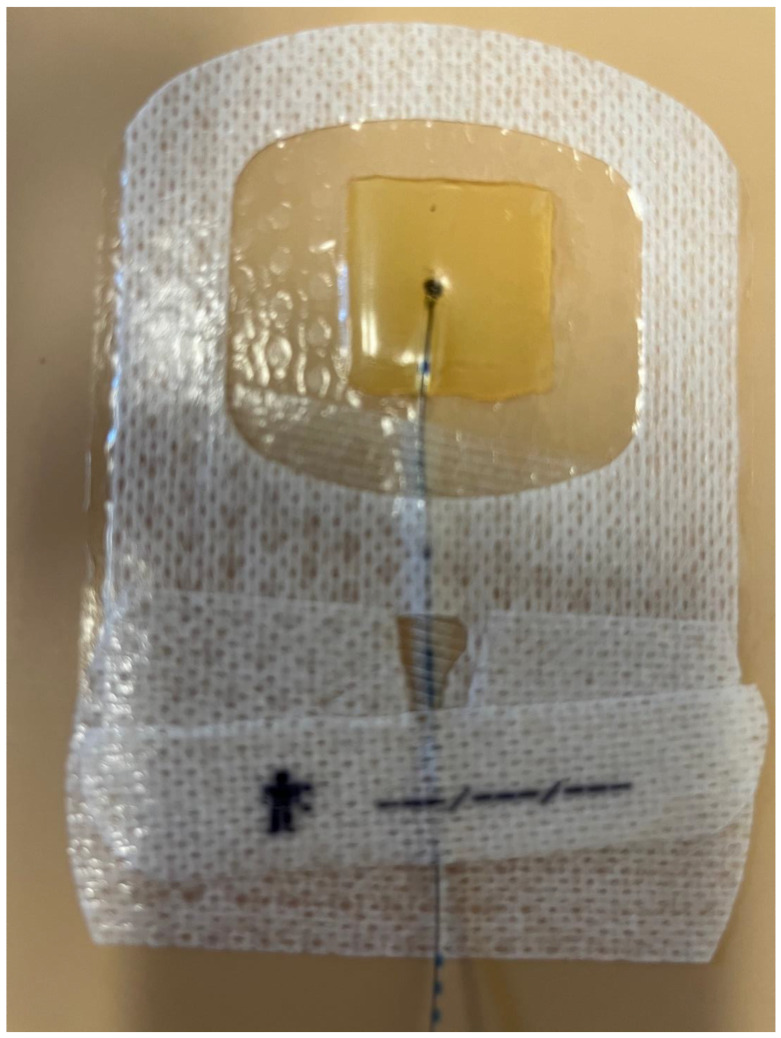
Securement dressing option that contains chlorhexidine gluconate and is transparent to allow visibility of the insertion site, called Tegaderm CHG Chlorhexidine Gluconate IV Securement Dressing (3M Tegaderm^TM^).

Despite prolonged catheter use, the infection rate remained low, in line with established infection prevention protocols. We had zero catheter-related infections or insertion site infections, which is lower than the rate published in the literature of 0.9% [2]. This reinforces the importance of maintaining strict aseptic techniques when managing PNCs. Our practice is to check the dressings for PNCs daily and monitor temperature and laboratory results. All nine of our patients were on antibiotics for most of the duration of the nerve block. In the available literature, antibiotics reduce the incidence of infection, and antibiotics are the treatment for catheter-related infections [14,15,16]. Additionally, we used an antimicrobial adjuvant in 24% of our catheter dressings (Biopatch^®^, Ethicon, Inc., Somerville, NJ, USA). See Figure 1 and Figure 2 for a detailed representation of the dressing techniques used in these cases. Although controversial, this is another method to reduce infections that was not used in any severe infection cases we reviewed in the literature. The Biopatch has been used in adult patients to minimize the infection rate of epidural catheters and in pediatric and adult patients to reduce the risk of infection of central venous catheters [19,20]. There is another securement dressing option that contains chlorhexidine gluconate and is transparent to allow visibility of the insertion site, called Tegaderm CHG Chlorhexidine Gluconate IV Securement Dressing (3M Tegaderm^TM^) (Figure 3). This device has also been used to reduce the risk of infection of indwelling catheters [21]. However, we did not use it in our patient population, and more studies need to be done to investigate the complication rates with this dressing technique. In addition to checking dressings, using antibiotics, and using a Biopatch, we also changed the dressing when it was contaminated and replaced and rotated sites for peripheral nerve catheters. 

Suppose nerve block catheters are placed with a strict aseptic technique, the site is tunneled or dressed with an antimicrobial dressing, and the site is monitored daily for infection. In that case, we believe it is safe to prolong the duration of these catheters for pediatric patients undergoing multiple surgeries. The risks of catheter site infection are also mitigated by monitoring the patient for fever and other systemic signs of infection and by having the patient on systemic antibiotics. 

We observed no infection and a low frequency of complications such as dressing contamination, catheter disconnect, and dislodgment. This suggests that long-term nerve block catheters can be considered for pain management in this patient population. The pediatric trauma patient population is at high risk for developing chronic pain after their injuries. We believe that our techniques and methods to reduce infection lead to a safe, beneficial pain management strategy that outweighs the risk of infectious related complications. We believe this will help pediatric trauma patients avoid the risk of chronic pain. We also believe that it will help avoid systemic opioids, which lead to many undesirable side effects, such as addiction, tolerance, dependence, constipation, and respiratory depression [17].

However, it is essential to acknowledge the limitations of this case series study. The sample size was small, and the results may not be generalizable to all pediatric trauma patients. Further research with larger sample sizes and randomized controlled trials would be valuable in confirming and expanding upon our findings.

In conclusion, our study provides evidence that a wide variety of nerve block catheters can be maintained for up to 15 days (about 2 weeks) without an increased risk of infection or local anesthetic toxicity if strict aseptic technique is used with daily monitoring of the site. 

The knowledge gained from this research can help pain doctors make decisions regarding maintaining these catheters for longer than 72 h (about 3 days). It can improve the quality of care for pediatric trauma patients that will have multiple surgeries and remain hospitalized for longer time frames.

## Figures and Tables

**Table 1:** Demographic data of patients receiving prolonged continuous nerve block catheters.

Patient #	Age (Year)	Weight (kg)	ASA * Class	Injury	Surgeries
1	8	36	2	Degloving of right lateral foot	Repeated irrigation, debridement, wound vacuum changes, and free flap skin graft surgery.
2	4	24	2	Lawnmower injury to right lower extremity	Repeated irrigation, debridement, wound vacuum changes, and free flap skin graft surgery.
3	3	20	2	Degloving injury from a lawnmower to bilateral lower extremities	Repeated irrigation and debridement, wound vacuum changes
4	3	18	3	Right upper extremity amputation from lawnmower	Irrigation and debridement, wound vacuum placement, complete amputation
5	12	52	1	Dirt bike injury causing left lower extremity avulsion	Irrigation and debridement
6	6	22	1	Bilateral trans metatarsal amputations from lawnmower injury	Repeated irrigation and debridement, wound vacuum placement, and changes
7	4	14	3	Lawnmower induced right lower extremity fractures, lacerations, degloving injury	Open reduction and internal fixation, exploration of wound, wound vacuum placement, and changes
8	6	21	3	Septic shock leading to extra-corporeal membrane support, complicated with thromboses to bilateral upper extremities	Irrigation and debridement to bilateral upper extremity, skin grafting
9	4	18	2	Partial amputation from lawn mower to left lower extremity	Left foot irrigation and debridement, wound vacuum changes

* ASA (American Society of Anesthesiologists) class refers to the Anesthesiology physical classification I–VI. ASA I refers to a normal healthy patient. ASA II refers to mild systemic disease. ASA III refers to severe systemic disease. ASA IV refers to systemic disease that is a constant threat to life. ASA V refers to a patient who is not expected to survive without surgery. ASA VI is a declared brain-dead patient for organ donation.

**Table 2:** The duration, location, and related data for nine patients receiving continuous peripheral nerve catheters. Each catheter is listed, with multiple catheters placed per patient #. There were no incidences of local anesthetic-related complications. No interventions were noted for any catheter-related complications. Normal white blood cell count is 5–17 × 10^9^/L.

Patient #	Nerve Block Location	# of Catheters	Duration of Catheters Individually	Total Number of Days with a Catheter in Place	Operating Room Antibiotics	Scheduled Antibiotics	Bio Patch Used	Tunneled Catheter	Catheter Complications	Fevers	Infections
1	Right popliteal	2	11, 11	22	Yes	Yes	Not noted	#2	Leaking day 11 of the first catheter	No	No
1	Epidural	1	7	7	Yes	No	Not noted	Not noted	None	No	No
2	Right femoral	4	11, 8, 4, 10	33	Yes	Yes	Not noted	Not noted	None	No	Leukocytosis
2	Right popliteal	2	11, 10	21	Yes	Yes *	Not noted	Not noted	Excoriation and peeling up from dressing on day 6	No	Leukocytosis
2	Right anterior sciatic	1	12	12	Yes	No, but it started during the catheter	Not noted	Not noted	None	No	Leukocytosis
2	Epidural	2	8, 6	14	Yes	Yes	Not noted	#1	None	No	Positive for Clostridium Difficile
3	Right popliteal	3	3, 8, 7		Yes	Yes	Not noted	Not noted	None	No	No
3	Left popliteal	3	3, 8, 7		Yes	Yes	Not noted	Not noted	None	No	No
3	Right sciatic	2	13, 10	23	Yes	Yes	Not noted	Not noted	None	No	No
3	Left sciatic	2	13, 10	23	Yes	Yes	Not noted	Not noted	Leaking of the second sciatic catheter on day 5, redressed, site clean	No	No
3	Epidural	1	8	8	Yes	Yes	Not noted	Yes	Redness day 6 at the insertion site, redressed; no abscess, not infected	No	No
4	Right supraclavicular	2	7, 3	10	Yes	Yes	Not noted	Not noted	The second catheter had a loose dressing and was removed early on day 3, and the site was clean.	Yes	No
4	Right infraclavicular	2	7, 2	9	Yes	Yes	Not noted	Not noted	The second catheter had leaked and was removed early; the site was clean.	No	No
5	Left popliteal	1	11	11	Yes	Yes	Not noted	Not noted	None	No	No
6	Left popliteal	2	11, 9	20	Yes	Yes	Not noted	Not noted	None	No	No
6	Right popliteal	3	3, 6, 9	18	Yes	Yes	Not noted	Not noted	One was accidentally removed, one had an unwitnessed disconnect and removed early, and fever for the third.	Yes	No
7	Right femoral	4	3, 15, 2, 3	23	Yes	Yes	Not noted	Not noted	Patient removed	No	No
7	Right sciatic	4	3, 7, 13, 5	28	Yes	Yes	Not noted	Not noted	Urine, stool contamination;	No	No
7	Right lumbar plexus	2	2, 3	5	Yes	Yes	Not noted	Not noted	Leaking, reinforced, removed early, site clean	No	No
7	Right popliteal	1	9	9	Yes	Yes	Yes	Not noted	Urinated, diarrhea	No	No
8	Left supraclavicular	2	14, 4	18	Yes	Yes	Yes	Not noted	None	Yes	No, leukocytosis, wound infection
8	Right supraclavicular	2	14, 7	21	Yes	Yes	Yes	Not noted	Second removed by accident early, site clean	Yes	No
9	Left sciatic	1	9	9	Yes	Yes	Yes	Not noted	Stool contamination	Yes	No
9	Left femoral	2	9, 3	12	Yes	Yes	Yes	Not noted	None	No	No
9	Left popliteal	1	11	11	Yes	Yes	Yes	Not noted	Disconnect/dressing came off, removed, site clean	No	No

* Antibiotics were completed before the nerve block was removed but were restarted by the surgical and trauma team due to leukocytosis.

## Data Availability

Data is unavailable due to privacy or ethical restrictions.
